# 4-[(4-Meth­oxy­benzyl­idene)amino]­benzene­sulfonamide

**DOI:** 10.1107/S1600536812018818

**Published:** 2012-05-02

**Authors:** Omoruyi G. Idemudia, Alexander P. Sadimenko, Anthony J. Afolayan, Eric C. Hosten

**Affiliations:** a Department of Chemistry, University of Fort Hare, Private Bag X1314, Alice 5700, South Africa; b Department of Chemistry, Nelson Mandela Metropolitan University, PO Box 77000, Port Elizabeth 6031, South Africa

## Abstract

The title Schiff base compound, C_14_H_14_N_2_O_3_S, is non-planar, with a dihedral angle of 24.16 (7)° between the benzene rings. In the crystal, N—H⋯O and N—H⋯N hydrogen bonds link the mol­ecules into a layer parallel to (011). Intra- and inter­layer C—H⋯O inter­actions and π–π inter­actions [centroid–centroid distances = 3.8900 (9) and 3.9355 (8) Å] are also present.

## Related literature

For general background to the applications of sulfanilamide Schiff bases, see: Gupta *et al.* (2003 ▶); Khalil *et al.* (2009 ▶); Nagpal & Singh (2004 ▶); Sharaby (2007 ▶); Wu *et al.* (2004 ▶).
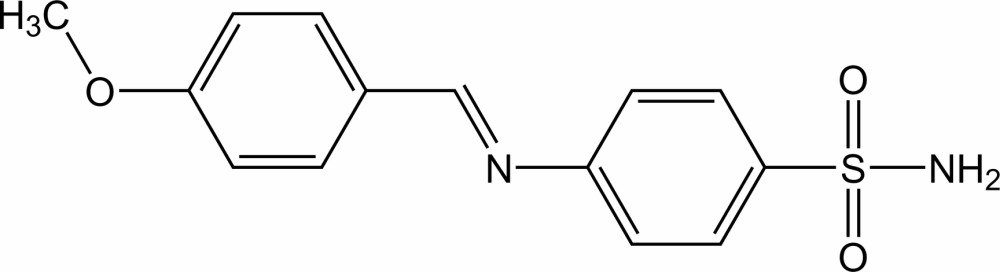


## Experimental

### 

#### Crystal data

C_14_H_14_N_2_O_3_S
*M* *_r_* = 290.33
Monoclinic, 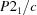

*a* = 16.3315 (5) Å
*b* = 11.1597 (3) Å
*c* = 7.6876 (3) Å
β = 100.661 (1)°
*V* = 1376.92 (8) Å^3^
*Z* = 4
Mo *K*α radiation
μ = 0.24 mm^−1^
*T* = 200 K
0.60 × 0.33 × 0.12 mm

#### Data collection

Bruker APEXII CCD diffractometer
Absorption correction: multi-scan (*SADABS*; Bruker, 2001 ▶) *T*_min_ = 0.90, *T*_max_ = 0.97
10501 measured reflections
3383 independent reflections
2821 reflections with *I* > 2σ(*I*)
*R*_int_ = 0.017

#### Refinement

*R*[*F* ^2^ > 2σ(*F* ^2^)] = 0.035
*wR*(*F* ^2^) = 0.095
*S* = 1.08
3383 reflections
182 parameters
H-atom parameters constrained
Δρ_max_ = 0.41 e Å^−3^
Δρ_min_ = −0.39 e Å^−3^

### 

Data collection: *APEX2* (Bruker, 2007 ▶); cell refinement: *SAINT* (Bruker, 2007 ▶); data reduction: *SAINT*; program(s) used to solve structure: *SHELXS97* (Sheldrick, 2008 ▶); program(s) used to refine structure: *SHELXL97* (Sheldrick, 2008 ▶); molecular graphics: *ORTEP-3* (Farrugia, 1997 ▶); software used to prepare material for publication: *PLATON* (Spek, 2009 ▶) and *publCIF* (Westrip, 2010 ▶).

## Supplementary Material

Crystal structure: contains datablock(s) global, I. DOI: 10.1107/S1600536812018818/hy2541sup1.cif

Structure factors: contains datablock(s) I. DOI: 10.1107/S1600536812018818/hy2541Isup2.hkl

Supplementary material file. DOI: 10.1107/S1600536812018818/hy2541Isup3.cdx

Supplementary material file. DOI: 10.1107/S1600536812018818/hy2541Isup4.cml

Additional supplementary materials:  crystallographic information; 3D view; checkCIF report

## Figures and Tables

**Fig. 1.**
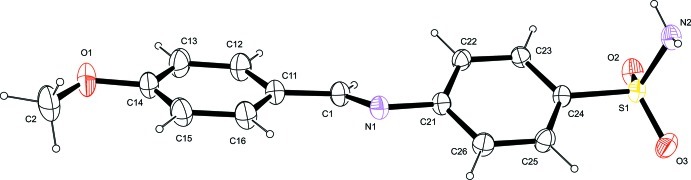
The molecular structure of the title compound, with displacement ellipsoids drawn at the 50% probability level.

**Fig. 2.**
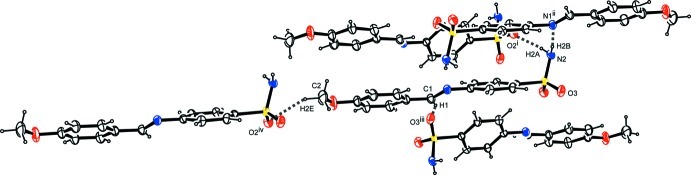
Hydrogen bonds in the title compound. [Symmetry codes: (i) x, -y+3/2, z-1/2; (ii) -x+1, -y+2, -z+1; (iii) -x+1, y-1/2, -z+3/2; (iv) x-1, y, z-1.]

**Fig. 3.**
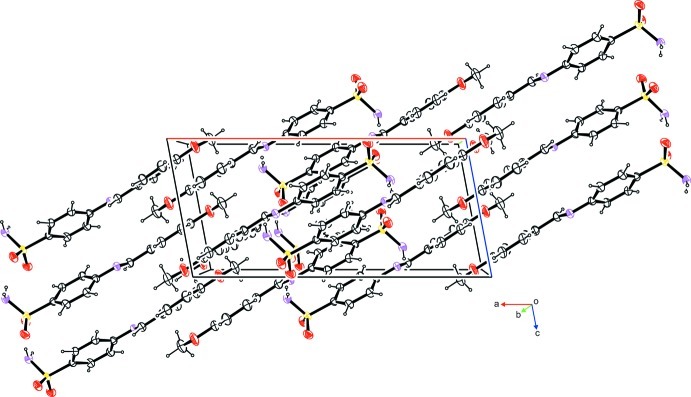
Crystal packing of the title compound viewed along [0 1 0].

## supplementary crystallographic information

### Comment

Biological applications of sulfa compounds either alone or as metal complexes are well known (Gupta *et al.*, 2003; Nagpal & Singh, 2004). Their chelating powers towards metal ions tend to increase on forming a Schiff base by way of reaction with a carbonyl (Khalil *et al.*, 2009; Sharaby, 2007; Wu *et al.*, 2004). Herein, we report a new sulfanilamide Schiff base (Fig. 1), as part of our look at developing better chelating ligands from biologically active amine compounds.

The least-squares planes through the phenyl rings of the benzenesulfonamide and methoxybenzaldehyde groups have a dihedral angle of 24.16 (7)°. In the crystal, the molecules are stacked along the *c* axis and linked by N—H···O and N—H···N hydrogen bonds (Table 1 and Fig. 2) into a layer parallel to (0 1 1) (Fig. 3). The least-squares planes through adjacent two methoxybenzaldehyde phenyl rings (C11–C16) are almost parallel with a dihedral angle of 3.97° and a centroid-to-centroid distance of 3.8900 (9) Å. The centroid-to-centroid distance between adjacent two benzenesulfonamide phenyl rings (C21–C26) is 3.9355 (8) Å. C22—H22···π interaction occurs with the adjacent C21–C26 ring (H···*Cg* distance = 2.81 Å). Intra- and interlayer C—H···O interactions are also observed.

### Experimental

A mixture of (4-aminobenzenesulfonamide)sulfadiazine and 4-methoxybenzaldehyde (anisaldehyde) (molar ratio 1:1) in methanol was refluxed for 15 h. The resultant pale yellow precipitate was isolated by filtration and recrystalized from methanol. Yield 68% and melting point 199–201°C. Single crystals suitable for X-ray analysis were obtained from methanol by slow evaporation at room temperature.

### Refinement

C-bound H atoms were placed in calculated positions and refined as riding atoms, with C—H = 0.95 (CH), 0.98 (CH_3_) Å and with *U*_iso_(H) = 1.2(1.5 for methyl)*U*_eq_(C). N-bound H atoms were located on a difference Fourier map and refined as riding with *U*_iso_(H) = 1.2*U*_eq_(N).

### Crystal data

**Table:**

C_14_H_14_N_2_O_3_S	*F*(000) = 608
*M**_r_* = 290.33	*D*_x_ = 1.401 Mg m^−^^3^
Monoclinic, *P*2_1_/*c*	Melting point: 473.15 K
Hall symbol: -P 2ybc	Mo *K*α radiation, λ = 0.71073 Å
*a* = 16.3315 (5) Å	Cell parameters from 118 reflections
*b* = 11.1597 (3) Å	θ = 3.1–29.3°
*c* = 7.6876 (3) Å	µ = 0.24 mm^−^^1^
β = 100.661 (1)°	*T* = 200 K
*V* = 1376.92 (8) Å^3^	Platelet, yellow
*Z* = 4	0.60 × 0.33 × 0.12 mm

### Data collection

**Table:**

Bruker APEXII CCD diffractometer	3383 independent reflections
Radiation source: sealed tube	2821 reflections with *I* > 2σ(*I*)
Graphite monochromator	*R*_int_ = 0.017
Detector resolution: 8.3333 pixels mm^-1^	θ_max_ = 28.3°, θ_min_ = 2.2°
φ and ω scans	*h* = −21→21
Absorption correction: multi-scan (*SADABS*; Bruker, 2001)	*k* = −14→14
*T*_min_ = 0.90, *T*_max_ = 0.97	*l* = −10→8
10501 measured reflections	

### Refinement

**Table:**

Refinement on *F*^2^	Primary atom site location: structure-invariant direct methods
Least-squares matrix: full	Secondary atom site location: difference Fourier map
*R*[*F*^2^ > 2σ(*F*^2^)] = 0.035	Hydrogen site location: inferred from neighbouring sites
*wR*(*F*^2^) = 0.095	H-atom parameters constrained
*S* = 1.08	*w* = 1/[σ^2^(*F*_o_^2^) + (0.0391*P*)^2^ + 0.7392*P*] where *P* = (*F*_o_^2^ + 2*F*_c_^2^)/3
3383 reflections	(Δ/σ)_max_ = 0.001
182 parameters	Δρ_max_ = 0.41 e Å^−^^3^
0 restraints	Δρ_min_ = −0.39 e Å^−^^3^

### Special details

**Table:**

Geometry. All e.s.d.'s (except the e.s.d. in the dihedral angle between two l.s. planes) are estimated using the full covariance matrix. The cell e.s.d.'s are taken into account individually in the estimation of e.s.d.'s in distances, angles and torsion angles; correlations between e.s.d.'s in cell parameters are only used when they are defined by crystal symmetry. An approximate (isotropic) treatment of cell e.s.d.'s is used for estimating e.s.d.'s involving l.s. planes.
Refinement. Refinement of *F*^2^ against ALL reflections. The weighted *R*-factor *wR* and goodness of fit *S* are based on *F*^2^, conventional *R*-factors *R* are based on *F*, with *F* set to zero for negative *F*^2^. The threshold expression of *F*^2^ > σ(*F*^2^) is used only for calculating *R*-factors(gt) *etc*. and is not relevant to the choice of reflections for refinement. *R*-factors based on *F*^2^ are statistically about twice as large as those based on *F*, and *R*- factors based on ALL data will be even larger.

### Fractional atomic coordinates and isotropic or equivalent isotropic displacement parameters (Å^2^)

**Table:**

	*x*	*y*	*z*	*U*_iso_*/*U*_eq_	
S1	0.663198 (19)	0.87003 (3)	0.83688 (5)	0.02211 (10)	
O1	−0.04751 (7)	0.67929 (13)	0.0325 (2)	0.0479 (4)	
O2	0.68589 (6)	0.74895 (9)	0.88958 (16)	0.0306 (3)	
O3	0.66935 (7)	0.96150 (10)	0.96956 (16)	0.0333 (3)	
N1	0.31032 (7)	0.85378 (10)	0.45630 (17)	0.0231 (2)	
N2	0.72148 (7)	0.90797 (11)	0.69963 (17)	0.0248 (3)	
H2A	0.719	0.8559	0.6172	0.03*	
H2B	0.7141	0.9792	0.6628	0.03*	
C1	0.27140 (8)	0.75396 (13)	0.4333 (2)	0.0250 (3)	
H1	0.2986	0.6843	0.4867	0.03*	
C2	−0.10221 (11)	0.7766 (2)	−0.0293 (3)	0.0553 (6)	
H2C	−0.0756	0.8292	−0.1044	0.083*	
H2D	−0.1142	0.8221	0.0721	0.083*	
H2E	−0.1543	0.7451	−0.0979	0.083*	
C11	0.18743 (8)	0.74102 (13)	0.3295 (2)	0.0257 (3)	
C12	0.15750 (9)	0.62521 (14)	0.2867 (2)	0.0319 (3)	
H12	0.1914	0.558	0.3275	0.038*	
C13	0.07929 (10)	0.60763 (15)	0.1859 (2)	0.0365 (4)	
H13	0.0598	0.5287	0.1558	0.044*	
C14	0.02902 (9)	0.70602 (16)	0.1285 (2)	0.0335 (4)	
C15	0.05726 (9)	0.82138 (15)	0.1709 (2)	0.0344 (4)	
H15	0.0228	0.8884	0.1321	0.041*	
C16	0.13644 (9)	0.83808 (14)	0.2707 (2)	0.0310 (3)	
H16	0.1561	0.9171	0.2993	0.037*	
C21	0.39382 (8)	0.85383 (12)	0.55004 (19)	0.0206 (3)	
C22	0.44881 (8)	0.75989 (12)	0.5357 (2)	0.0225 (3)	
H22	0.43	0.6923	0.4643	0.027*	
C23	0.53057 (8)	0.76496 (12)	0.6253 (2)	0.0223 (3)	
H23	0.5675	0.7002	0.6176	0.027*	
C24	0.55836 (8)	0.86504 (11)	0.72618 (19)	0.0199 (3)	
C25	0.50485 (9)	0.96019 (12)	0.7391 (2)	0.0244 (3)	
H25	0.5244	1.0287	0.8077	0.029*	
C26	0.42248 (8)	0.95441 (12)	0.6508 (2)	0.0246 (3)	
H26	0.3857	1.0192	0.6592	0.03*	

### Atomic displacement parameters (Å^2^)

**Table:**

	*U*^11^	*U*^22^	*U*^33^	*U*^12^	*U*^13^	*U*^23^
S1	0.01897 (16)	0.02111 (17)	0.02420 (19)	−0.00156 (11)	−0.00138 (12)	0.00123 (13)
O1	0.0217 (5)	0.0634 (9)	0.0533 (9)	−0.0056 (5)	−0.0072 (5)	−0.0087 (7)
O2	0.0250 (5)	0.0263 (5)	0.0377 (7)	0.0009 (4)	−0.0015 (4)	0.0113 (5)
O3	0.0301 (5)	0.0363 (6)	0.0304 (6)	−0.0027 (4)	−0.0023 (4)	−0.0097 (5)
N1	0.0194 (5)	0.0234 (6)	0.0252 (6)	0.0002 (4)	0.0007 (4)	0.0015 (5)
N2	0.0224 (5)	0.0197 (5)	0.0314 (7)	−0.0027 (4)	0.0029 (5)	0.0025 (5)
C1	0.0216 (6)	0.0235 (6)	0.0284 (8)	−0.0005 (5)	0.0010 (5)	0.0029 (5)
C2	0.0239 (8)	0.0838 (16)	0.0532 (13)	0.0076 (9)	−0.0063 (8)	−0.0003 (12)
C11	0.0198 (6)	0.0279 (7)	0.0282 (8)	−0.0023 (5)	0.0013 (5)	0.0010 (6)
C12	0.0263 (7)	0.0282 (7)	0.0391 (9)	−0.0030 (6)	0.0005 (6)	0.0016 (7)
C13	0.0282 (7)	0.0346 (8)	0.0440 (10)	−0.0092 (6)	−0.0002 (7)	−0.0053 (7)
C14	0.0188 (6)	0.0477 (9)	0.0323 (9)	−0.0032 (6)	0.0005 (6)	−0.0036 (7)
C15	0.0226 (7)	0.0388 (9)	0.0398 (10)	0.0041 (6)	0.0008 (6)	0.0013 (7)
C16	0.0234 (7)	0.0292 (7)	0.0388 (9)	−0.0004 (6)	0.0014 (6)	0.0009 (7)
C21	0.0191 (6)	0.0199 (6)	0.0217 (7)	−0.0003 (5)	0.0013 (5)	0.0036 (5)
C22	0.0218 (6)	0.0191 (6)	0.0259 (7)	−0.0024 (5)	0.0026 (5)	−0.0029 (5)
C23	0.0202 (6)	0.0193 (6)	0.0274 (7)	0.0002 (5)	0.0045 (5)	−0.0008 (5)
C24	0.0183 (5)	0.0192 (6)	0.0213 (7)	−0.0016 (4)	0.0014 (5)	0.0022 (5)
C25	0.0254 (6)	0.0176 (6)	0.0281 (7)	−0.0009 (5)	0.0000 (5)	−0.0023 (5)
C26	0.0238 (6)	0.0190 (6)	0.0297 (8)	0.0026 (5)	0.0015 (5)	−0.0011 (5)

### Geometric parameters (Å, º)

**Table:**

S1—O3	1.4333 (11)	C12—H12	0.95
S1—O2	1.4393 (10)	C13—C14	1.393 (2)
S1—N2	1.6032 (13)	C13—H13	0.95
S1—C24	1.7663 (13)	C14—C15	1.386 (2)
O1—C14	1.3617 (17)	C15—C16	1.389 (2)
O1—C2	1.430 (2)	C15—H15	0.95
N1—C1	1.2786 (18)	C16—H16	0.95
N1—C21	1.4196 (16)	C21—C26	1.3945 (19)
N2—H2A	0.8551	C21—C22	1.3978 (18)
N2—H2B	0.8452	C22—C23	1.3856 (18)
C1—C11	1.4607 (18)	C22—H22	0.95
C1—H1	0.95	C23—C24	1.3875 (19)
C2—H2C	0.98	C23—H23	0.95
C2—H2D	0.98	C24—C25	1.3904 (18)
C2—H2E	0.98	C25—C26	1.3916 (19)
C11—C16	1.390 (2)	C25—H25	0.95
C11—C12	1.399 (2)	C26—H26	0.95
C12—C13	1.380 (2)		
			
O3—S1—O2	119.20 (7)	C14—C13—H13	120.1
O3—S1—N2	107.96 (7)	O1—C14—C15	124.27 (15)
O2—S1—N2	106.25 (7)	O1—C14—C13	115.28 (15)
O3—S1—C24	107.42 (6)	C15—C14—C13	120.44 (14)
O2—S1—C24	106.39 (6)	C14—C15—C16	119.35 (15)
N2—S1—C24	109.38 (6)	C14—C15—H15	120.3
C14—O1—C2	117.90 (15)	C16—C15—H15	120.3
C1—N1—C21	118.48 (12)	C15—C16—C11	121.04 (15)
S1—N2—H2A	110.9	C15—C16—H16	119.5
S1—N2—H2B	113.8	C11—C16—H16	119.5
H2A—N2—H2B	114.0	C26—C21—C22	119.53 (12)
N1—C1—C11	123.66 (13)	C26—C21—N1	118.32 (12)
N1—C1—H1	118.2	C22—C21—N1	122.04 (12)
C11—C1—H1	118.2	C23—C22—C21	120.29 (12)
O1—C2—H2C	109.5	C23—C22—H22	119.9
O1—C2—H2D	109.5	C21—C22—H22	119.9
H2C—C2—H2D	109.5	C22—C23—C24	119.74 (12)
O1—C2—H2E	109.5	C22—C23—H23	120.1
H2C—C2—H2E	109.5	C24—C23—H23	120.1
H2D—C2—H2E	109.5	C23—C24—C25	120.64 (12)
C16—C11—C12	118.77 (13)	C23—C24—S1	118.85 (10)
C16—C11—C1	123.09 (13)	C25—C24—S1	120.51 (10)
C12—C11—C1	118.13 (13)	C24—C25—C26	119.57 (13)
C13—C12—C11	120.67 (14)	C24—C25—H25	120.2
C13—C12—H12	119.7	C26—C25—H25	120.2
C11—C12—H12	119.7	C25—C26—C21	120.19 (12)
C12—C13—C14	119.73 (15)	C25—C26—H26	119.9
C12—C13—H13	120.1	C21—C26—H26	119.9
			
C21—N1—C1—C11	−175.87 (13)	C26—C21—C22—C23	1.9 (2)
N1—C1—C11—C16	−11.2 (2)	N1—C21—C22—C23	178.05 (13)
N1—C1—C11—C12	168.42 (16)	C21—C22—C23—C24	−1.5 (2)
C16—C11—C12—C13	0.9 (3)	C22—C23—C24—C25	0.3 (2)
C1—C11—C12—C13	−178.67 (16)	C22—C23—C24—S1	−179.27 (11)
C11—C12—C13—C14	−1.1 (3)	O3—S1—C24—C23	−163.03 (12)
C2—O1—C14—C15	0.3 (3)	O2—S1—C24—C23	−34.34 (13)
C2—O1—C14—C13	179.64 (17)	N2—S1—C24—C23	80.04 (12)
C12—C13—C14—O1	−178.90 (16)	O3—S1—C24—C25	17.41 (14)
C12—C13—C14—C15	0.4 (3)	O2—S1—C24—C25	146.11 (12)
O1—C14—C15—C16	179.57 (16)	N2—S1—C24—C25	−99.52 (13)
C13—C14—C15—C16	0.3 (3)	C23—C24—C25—C26	0.5 (2)
C14—C15—C16—C11	−0.4 (3)	S1—C24—C25—C26	180.00 (11)
C12—C11—C16—C15	−0.2 (2)	C24—C25—C26—C21	0.0 (2)
C1—C11—C16—C15	179.39 (15)	C22—C21—C26—C25	−1.2 (2)
C1—N1—C21—C26	−148.41 (14)	N1—C21—C26—C25	−177.44 (13)
C1—N1—C21—C22	35.4 (2)		

### Hydrogen-bond geometry (Å, º)

**Table:**

*D*—H···*A*	*D*—H	H···*A*	*D*···*A*	*D*—H···*A*
N2—H2*A*···O2^i^	0.86	2.09	2.9280 (17)	166
N2—H2*B*···N1^ii^	0.85	2.08	2.9233 (17)	173
C1—H1···O3^iii^	0.95	2.55	3.4466 (18)	157
C2—H2*E*···O2^iv^	0.98	2.59	3.415 (2)	141

Symmetry codes: (i) *x*, −*y*+3/2, *z*−1/2; (ii) −*x*+1, −*y*+2, −*z*+1; (iii) −*x*+1, *y*−1/2, −*z*+3/2; (iv) *x*−1, *y*, *z*−1.
